# Tucuma Fat-Based Nanostructured Lipid Carriers: Physical Stability and Antifungal Activity

**DOI:** 10.1021/acsomega.5c11826

**Published:** 2026-05-06

**Authors:** Antônio Taylon Aguiar Gomes, Rayanne Rocha Pereira, Lindalva Maria de Meneses Costa Ferreira, Naila Ferreira da Cruz, Desireé Gyles Lynch, Patrícia Fagundes da Costa, Claudio Guedes Salgado, José Otávio Carréra Silva-Júnior, Roseane Maria Ribeiro-Costa

**Affiliations:** † Institute of Health Sciences, 37871 Federal University of Pará, Belém, Pará 66075-110, Brazil; ‡ Institute of Collective Health, Federal University of Western Pará, Santarém, Pará 68035-110, Brazil; § Institute of Biological Sciences, Federal University of Pará, Belém, Pará 66075-110, Brazil; ∥ College of Health Sciences, School of Pharmacy, 41642 University of Technology, Jamaica, Kingston 6, Jamaica

## Abstract

This study aimed to obtain and characterize NLCs from tucuma fat and evaluate them against fungal cultures. The NLCs were obtained by high-pressure homogenization of tucuma fat and carnauba wax as the oil phase and were evaluated for encapsulation efficiency (EE), drug loading (DL), zeta potential, particle size, and in vitro release kinetics over a 70-day period. Four formulations (NLC_1_, NLC_2_, NLC_3_, and NLC_4_) were prepared, showing particle size variation between 109.7 and 241.1 nm, PDI ranging from 0.16 to 0.41, and zeta potential between −12.7 mW and −27.5 mW. Only two of the four formulations were loaded with ketoconazole, presenting encapsulation efficiencies between 43.55% (NLC3) and 80.97% (NLC4), while drug loading values ranged from 2.18% (NLC_3_) to 4.03% (NLC_4_). After 70 days of storage at 8 and 28 °C, they maintained their physical characteristics (particle size, zeta potential, and PDI). The release kinetics of NLC_3_ and NLC4 were explained by the Korsmeyer-Peppas mathematical model. Finally, the antifungal activity of ketoconazole was improved 8-fold in NLC_3_ and NLC_4_. Finally, tucuma NLCs showed physical stability and biological efficacy.

## Graphical abstract

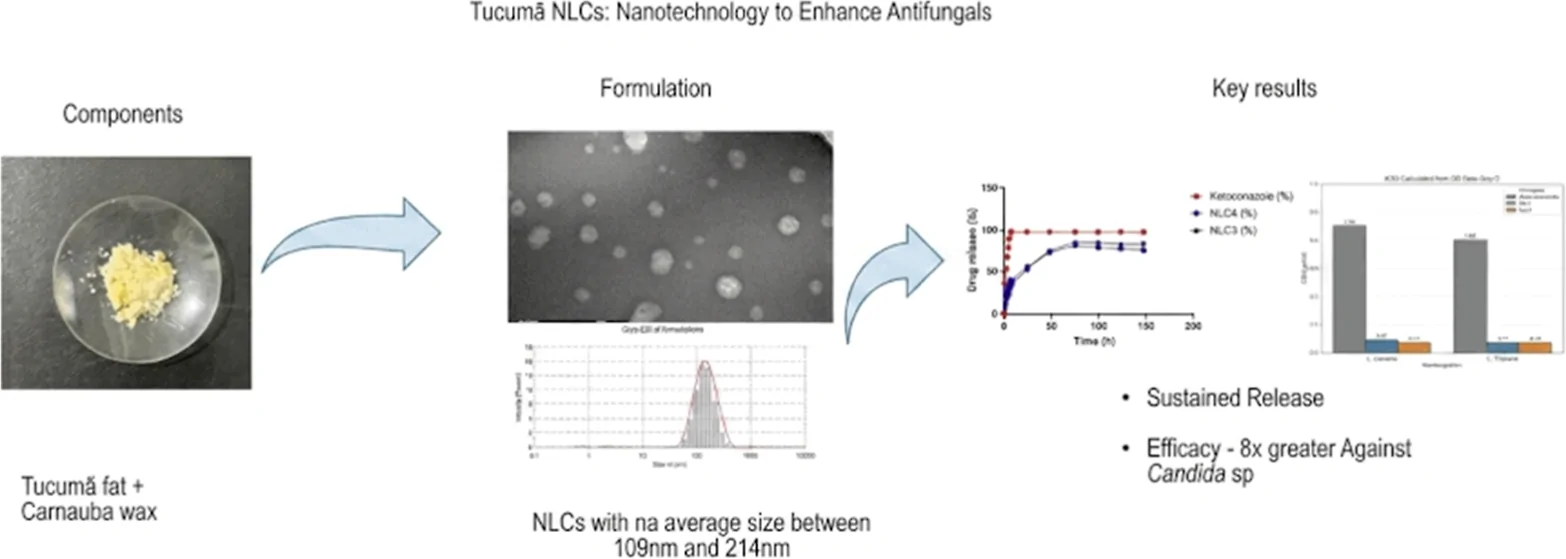

## 1 Introduction

The rise in fungal infections and increasing resistance to antifungal drugs are becoming a major global health challenge, also representing a significant economic burden. Each year, fungal infections lead to more than 1.5 million deaths worldwide.^1,2^ In this context, *Candida albicans* is known as the most prevalent opportunistic fungus in humans, responsible for 60% of mucosal infections and 40% of candidemia cases. Several virulence characteristics are known to contribute to infections caused by *C. albicans*, such as the ability to adhere to the host and nonliving medical surfaces, biofilm formation, and the production of hydrolytic enzymes. These infections are generally treated with azoles, polyenes, and echinocandins. However, antifungal drugs have limitations related to toxicity, recurrence of infections, and the development of drug resistance. Nanotechnology has been used to overcome these challenges.^3–5^

Nanotechnology has been a strategy to overcome the limitations associated with conventional methods of drug delivery.^6^ Nanocarriers are colloidal systems characterized by singular properties, including a high surface area-to-volume ratio, which confers significant advantages for pharmaceutical applications. The physicochemical properties of these systems—such as composition, morphology, surface charge, and functionalization—play a determinant role in their biological performance, influencing parameters such as stability, bioavailability, release profile, and therapeutic targeting. In this context, nanostructured lipid carriers (NLCs) represent a promising approach in the development of advanced drug delivery systems. However, their translation into clinical practice remains limited by relevant challenges, including complexity in formulation and scale-up processes, physicochemical instability during storage and transport, as well as biological barriers that may compromise biodistribution and therapeutic efficacy.^6,7^

The composition of NLCs is a tool to circumvent these problems. NLCs have a lipid core composed of a mixture of solid lipids and liquid lipids, remains solid at body temperature and room temperature. The mixing lipids forming an imperfect crystal, creating structural voids that enhance drug entrapments.^8,9^ In addition, the lipid core, improve the NLC stability in long-term storage, because the liquid lipids, produce less ordered crystal lattice which results in more stable NLC formulations, avoiding expulsion of the incorporated drug.^10–12^

Additionally, The lipid core is composed of biocompatible lipids that ensure tolerability and safety. Vegetable fats are a natural blend of liquid and solid lipids composed of saturated and unsaturated fatty acids and, moreover, they exhibit several biological effects.^13^ Lauric, myristic, and palmitic acids have antimicrobial effects,^14,15^ oleic acid also exhibits antimicrobial activity^15^ and functions as a skin penetration enhancer.^16,17^ In the fat extracted from the endocarp of the tucuma plant (*Astrocaryum vulgare* Mart.), lauric acid (47.89%), myristic acid (23.75%), oleic acid (12.00%) and palmitic acid (6.29%) are in the highest proportion among the fatty acids. This mixture of lauric acid (C12:0), myristic acid (C14:0), oleic acid (C18:1) and palmitic acid (6.29%) gives tucuma fat a melting point of around 34.74 °C.^18^

Although vegetable fats are biocompatible, particularly due to the biological properties of their fatty acids, the development of nanoparticles containing vegetable oils or fats presents several challenges.^19^ Regarding NLCs, these challenges translate into formulation development. The questions are how will this fat affect the crystallinity of the nanoparticles and, consequently, their physicochemical and biological characteristics. Previously, our research group worked on preformulation studies of tucuma fat, conducting chemical characterization studies and predictive analysis of its use as a raw material for nanostructured lipid nanoparticles (NLCs) in the pharmaceutical industry. These studies led us to add carnauba wax to tucuma fat at a 1:1 ratio (tucumã fat/carnauba wax), in an attempt to obtain a sufficiently crystalline lipid matrix for the production of NLCs.^18^

Our proposal is to construct a new bioactive lipid platform based on tucuma fat (*A. vulgare*) and carnauba wax loaded with ketoconazole. We hypothesize that the physicochemical and biological properties of tucuma fat make it a suitable excipient for ketoconazole-loaded NLCs. We produced ketoconazole-loaded tucuma NLCs and tested the ability of these formulations to transport ketoconazole. To this end, we evaluated their physicochemical characteristics, physical stability, morphology, and efficacy against *C. albicans* and *Candida tropicalis* strains.

## 2 Materials and Method

Tucuma fat was supplied by Amazon Oil Industry (Ananindeua, Pará, Brazil), Lot 001/2015. The sample was stored in plastic packaging and kept refrigerated at 8 °C until use. Carnauba wax was purchased from GM ceras (São Paulo, Brazil), Tween 80 was purchased from Labsynth (São Paulo, Brazil), and Pluronic F127 was purchased from Sigma-Aldrich (St. Louis, MO, USA). Ketoconazole was purchased from Natural Pharma (São Paulo, Brazil). The quality control reports, containing information on purity and other physical characteristics, are provided in Supporting Information 1.

### 2.1 NLC Preparation

The preparation of NLCs was carried out in two stages. In the first stage, a hot emulsion was produced using magnetic stirring to emulsify the aqueous and oil phases, and, in the second stage, this same emulsion was subjected to high-pressure homogenization (HPH). The NLCs were produced using the following procedure: the oil phase, composed of tucuma butter, carnauba wax, and a mixture of Tween 80 and Pluronic F-27 (1:1) was heated to 90 °C. The water was heated to the same temperature. After the oil phase was completely melted and homogenized, the aqueous phase was poured over the oil phase and kept under magnetic stirring for 10 min. The temperature was maintained and the first emulsion (or pre-emulsion) was subjected, to homogenization under high pressure (APV-1000, Albertslund, Denmark) at 600 bar for 3 cycles.^18^ A total of 4 formulations were produced (Table 1). All formulations were made for a final volume of 100 mL. In a previous study, we used an experimental design 2^2^ to evaluate the preparation conditions of tucumã NLCs. Specifically; we investigated how the concentration of surfactant and lipid phase influences PDI, droplet size, and zeta potential. The results of the DOE allowed us to conclude that NLCs containing 8%–15% lipids and 5% surfactant exhibited smaller particle size, lower PDI, and higher zeta potential values.^18^ Based on this study, we defined the composition shown in Table 1.

**Table 1:** Composition of NLC

samples	tucuma fat (%w/w)	carnauba wax (%w/w)	pluronic F-127 (%w/w)	tween 80 (%w/w)	ketoconazole (%w/w)	ultrapure water^a^
NLC_1_	7.500	7.500	2.5	2.5	0.00	80
NLC_2_	4.000	4.000	2.5	2.5	0.00	87
NLC_3_	7.125^a^	7.125	2.5	2.5	0.75	80
NLC_4_	3.8^a^	3.8	2.5	2.5	0.40	87

a Values calculated considering the qsf (quantity sufficient for).

### 2.2 Physicochemical Characterization

#### 2.2.1 Particle Size, Polydispersity Index PDI and Zeta Potential

All formulations were analyzed for particle size, polydispersity index (PDI), and zeta potential using a Zetasizer Nano ZS (Malvern Panalytical, Malvern, UK). Samples were diluted 1:100 in ultrapure water and analyzed at 25 °C. Measurements were performed in triplicate. The particle size was performed applying non-invasive back-scatter (scattering angle 173°).

#### 2.2.2 Encapsulation Efficiency and Drug Loading

The encapsulation efficiency (EE %) and drug loading (DL %) were performed by high-performance liquid chromatography (HPLC) coupled to a diode array detector (HPLC waters e2695 and DAD detector waters 2998), using an RP 18 column (5 μm particles, 3.9 × 150 mm) as the stationary phase, and a mixture of water and methanol (50:50) as the mobile phase under a flow rate of 1 mL/min, and the wavelength was 240 nm. The analytical curve was prepared with ketoconazole concentrations ranging from 0.25 to 20 μg/mL (equation of the line *y* = 43011.4252*x* + 568.6122, *R* ^2^ = 0.999). The encapsulation efficiency (EE) of the nanoparticles was determined by an indirect method. In a Falcon tube, 300 μL of NLC was transferred and 9.7 mL of methanol was added, vortexing for 1 min, followed by an ultrasonic bath (CD-4820, Kondentech, São Carlos, SP, Brazil) for 1 h. Then, it was centrifuged at 10 °C at 10,000 rpm for 30 min 100 μL of the supernatant was collected, 900 μL of mobile phase was added and analysis was performed on the HPLC. The EE % and DL % were calculated according to eqs 1 and 2, respectively.^20^

1 $$EE\left(\%\right)=\frac{Mtotal-Mfree}{Mtotal}\times 100$$

2 $$DL\left(\%\right)=\frac{Mtotal-Mfree}{Mlipid}\times 10$$

#### 2.2.3 Stability Testing

The physical stability of NLCs was investigated by monitoring the encapsulation efficiency (%EE), drug carrying capacity (%DL), PDI, particle size and zeta potential of the formulations. The formulations were stored in three environments with different temperatures: 8 ± 5 °C (refrigerator), 25 ± 5 °C (room temperature) and 40 ± 5 °C (oven). The samples were monitored in triplicate for 70 days, performed on days 0, 7, 14, 21, 28, 35, 42, 49, 56, 63 and 70.^21^

#### 2.2.4 Transmission Electron Microscopy

The morphological characterization of tucumã NLCs was performed by transmission electron microscopy (JEOL, JEM-2100, Tokyo, Japan) using the negative staining technique. The NLC dispersions were diluted at a ratio of 1:1000, and a drop of the diluted sample was placed onto a 200-mesh copper grid. Excess liquid was carefully removed using filter paper. After a 5 min incubation period, a drop of 2% uranyl acetate solution in ethanol was applied to the grid for contrast enhancement. The samples were then air-dried at room temperature (20 °C) prior to analysis.

#### 2.2.5 X-ray Diffraction

X-ray diffraction (XRD) analysis of the NLC formulation was carried out using a MiniFlex X-ray diffractometer (Rigaku, Tokyo, Japan) equipped with Cu Kα radiation (λ = 1.5418 Å), operated at 30 kV. Approximately 200 mg of the air-dried NLC formulation was placed into the sample holder until completely filled and subsequently compressed with a glass slide to obtain a flat and uniform surface. The samples were scanned over a θ range from 2° to 35° at a scanning rate of 1.5° min^–1^, using a step size of 0.5°.

#### 2.2.6 In Vitro Release Study

The in vitro release kinetics of ketoconazole from NLCs were studied using the dialysis method. Formulations NLC_3_, NLC_4_, and an ethanolic solution of ketoconazole (0.75 mg/mL) were analyzed. The dialysis membrane (MWCO 12,000 to 14,000 Da, Sigma-Aldrich St. Louis, MO, USA) containing 1 mL of sample was placed in a beaker with 100 mL of the release medium, consisting of water: ethanol (50:50, v/v), at 37 °C and constant stirring at 100 rpm. At predetermined time intervals (0–140 h), 300 μL of the release medium was removed and replaced with the same volume of fresh medium. The ketoconazole concentration was evaluated by RP-HPLC using the method described in Section 2.3.2. The data were analyzed using mathematical modeling with KinetDS software (version 3.0, Krakow, Poland). The model that best described the release profile was selected based on the correlation coefficient (*R* ^2^).^20^

### 2.3 Determination of Antifungal Activity In Vitro

#### 2.3.1 Determination of Minimum Inhibitory Concentration

The minimum inhibitory concentration (MIC) against *C. albicans* and *C. tropicalis* strains was performed according to recommendations described in CLSI M27-A2 (NCCLS, 2002).^22^ Suspensions of *C. albicans* (INCQS 40175) and *C. tropicalis* (CBS 7987) were prepared from isolates grown for 24 h on potato dextrose agar. The colonies were suspended in 5 mL of sterile 0.145 mol/L saline (8.5 g/L NaCl; 0.85% saline) and adjusted in a spectrophotometer (FLUOstar Omega, BMG LABTECH) at 530 nm to optical densities (OD) of 0.09 to 0.13. This procedure provides a standard yeast suspension containing 1 × 10^6^ to 5 × 10^6^ cells per mL. The working suspension was produced from a 1:100 dilution, followed by a 1:20 dilution of the standard suspension with RPMI 1640 liquid medium, resulting in a concentration of 5.0 × 10^2^ to 2.5 × 10^3^ cells per mL. The drug samples ketoconazole, NLC_3_, and NLC_4_ were distributed in 96-well microdilution plates at concentrations of 0.063 to 16 μg/mL. The microdilution plates were incubated at 35 °C and visually examined after 24 and 72 h, and readings were performed on a microplate reader (FLUOstar Omega, BMG LABTECH).

#### 2.3.2 IC_50_ Determination

The IC_50_ parameter (50% inhibitory concentration) was determined from the raw optical density (OD) data obtained after 72 h of incubation (Day 3). The calculation followed the following mathematical and statistical steps: For each tested concentration, the absorbance reading of the sample was converted into a growth fraction using the eq 3

3 $$growth\;fraction\frac{ODsample-ODnegative\;control}{ODpositive\;control-ODnegative\;control}$$

where a value of 1.0 represents maximum growth (equal to the positive control) and 0.0 represents total inhibition.

Based on the growth fraction results, the exact IC_50_ value was calculated by linear interpolation between the logarithms of the two adjacent concentrations that crossed the 50% inhibition threshold.

#### 2.3.3 Determination of Minimum Fungicidal Concentration

The minimum fungicidal concentration of ketoconazole against *C. albicans* and *C. tropicalis* was determined by determining the minimum fungicidal concentration (MFC). A 20 μL aliquot was removed from each well of the microdilution plate and placed in a 150 mm Petri dish, then incubated at 32–35 °C for 24 h. The MFC was defined as the lowest concentration of ketoconazole capable of inhibiting fungal growth.

### 2.4 Statistical Analisys

The results were evaluated and expressed as mean ± standard deviation, using excel office 365 (2010). All measurements were performed in triplicate. The data obtained in the antifungal studies and stability studies were analyzed using software GraphPad 5.0 (RRID: SCR_002798). The stability data were analyzed by studying the correlation between the variables *x* and *y*. Analysis of variance (ANOVA) and Tukey’s test were used, considering significant results with *p* < 0.05 and *p* < 0.01.

## 3 Results

### 3.1 Physical Characterization and Stability Assessment of NLCs

The size of the NLCs ranged from 109.10 to 241.10 nm. Formulations with a higher percentage of lipid phase (NLC_1_ and NLC_3_) showed larger particle sizes (Table 2), however, this increase in size due to the increase in the oil phase was not statistically significant (*p* > 0.05) (Table 2).

**Table 2:** Physical Characteristics NLCa^a^

NLC tucuma	lipid phase (%)	size (nm)	PDI	zeta potential (mW)	EE (%)	DL (%)
NLC1	15	241.1	0.41	–24.10	-	-
NLC2	8	128.5	0.16	–19.6	-	-
NLC3	15	160.2	0.26	–12.7	43.55	2.18
NLC4	8	109.7	0.20	–27.5	80.97	4.03

a There was no significant difference (*p* > 0.05) with the increase in the oily phase, or with the incorporation of ketoconazole in the formulations.

The PDI of the formulations showed values of 0.16–0.41, while the zeta potential ranged from −12.7 mW to −27.5 mW. Similar to what occurred with particle size, we investigated whether increasing the oil phase concentration influences these parameters. Again, increasing the oil phase concentration in ketoconazole-loaded NLC_3_ and NLC_4_ did not significantly affect the zeta potential and PDI values (*p* > 0.05) (Table 2).

It was also verified whether the addition of ketoconazole to the NLC formulation altered its physical characteristics. The comparison of the formulations with and without ketoconazole was evaluated using two-way ANOVA, and no significant differences (*p* > 0.05) were observed for the three parameters tested: size, PDI, and zeta potential (Table 2). Regarding EE, NLC_3_ was 43.55% and 80.97% for NLC_4_. The drug loading was 2.18% for NLC_3_ and 4.03% for NLC_4_.

The physical measurements determined from the NLC stability study, carried out during 70 days, in which the samples were stored in three environments with different temperatures (8 °C ± 5 °C, 40 ± 5 °C and 25 ± 5 °C), had their particle size, zeta potential and PDI values measured (Figure 1). The formulations did not present significant changes (*p* > 0.05) in their physical parameters during the study period. NLC_1_ is the formulation has the highest PDI value; in Figure 1, it is possible to see how the PDI values of this formulation fluctuated between 0.4 and 0.6, reaching peaks of 0.6 (Figure 1).

**Figure 1:**
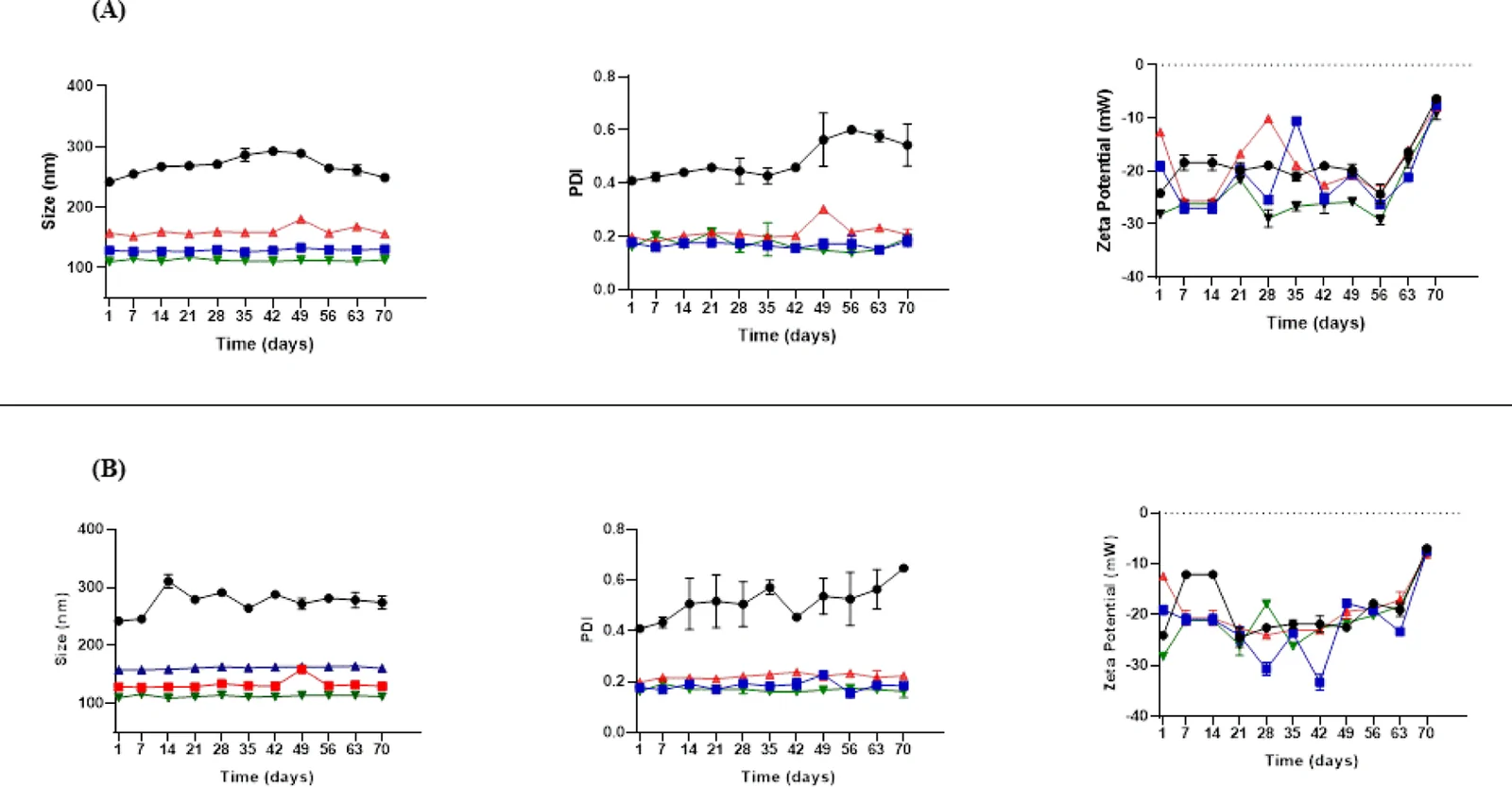
(A) Particle size, PDI size and zeta potential of NLC_1_(●), NLC_2_(■(red square)), NLC_3_(▲(blue triangle)) and NLC_4_(▼(green inverted triangle)) stored at 25 ± 5 °C. (B) Particle size, PDI size and zeta potential of NLC_1_(●), NLC_2_(■(red square)), NLC_3_(▲(blue triangle)) and NLC_4_(▼(green inverted triangle)) stored at 4 °C.

The formulations stored at 40 °C were unable to maintain stability for more than 7 days. On the seventh day after preparation, the PDI, zeta potential, and size measurements maintained the values observed at 24 h (Table 2); however, phase separation occurred on the following days.

With regard to stability, DL (%) and EE (%) were also monitored parameters (Figure 2A,B). Regarding DL (%), the values decreased throughout the storage period, and from the 14th day onward, there was a 1.0% decrease in values in both formulations stored under different temperature conditions. There was no decrease beyond the 1.0% observed after the 14th day (Figure 2A).About EE (%), the values also decreased for both formulations loaded with ketoconazole (NLC_3_ and NLC_4_) (Figure 2B). NLC_3_ showed a 10% decrease in the EE (%) value, recorded on the 14th day. After this period, no significant changes in the EE (%) value were recorded, this result was the same at both storage temperatures (8 and 25 °C). NLC_4_ showed a 30% decrease in the EE (%) value under both temperature conditions (8 and 25 °C) (Figure 2B). The significant variable for the decrease in EE (%) values in both formulations was storage time (Figure 2B). In other words, the decrease in EE (%) values was influenced by storage time, not by storage temperature.

**Figure 2:**
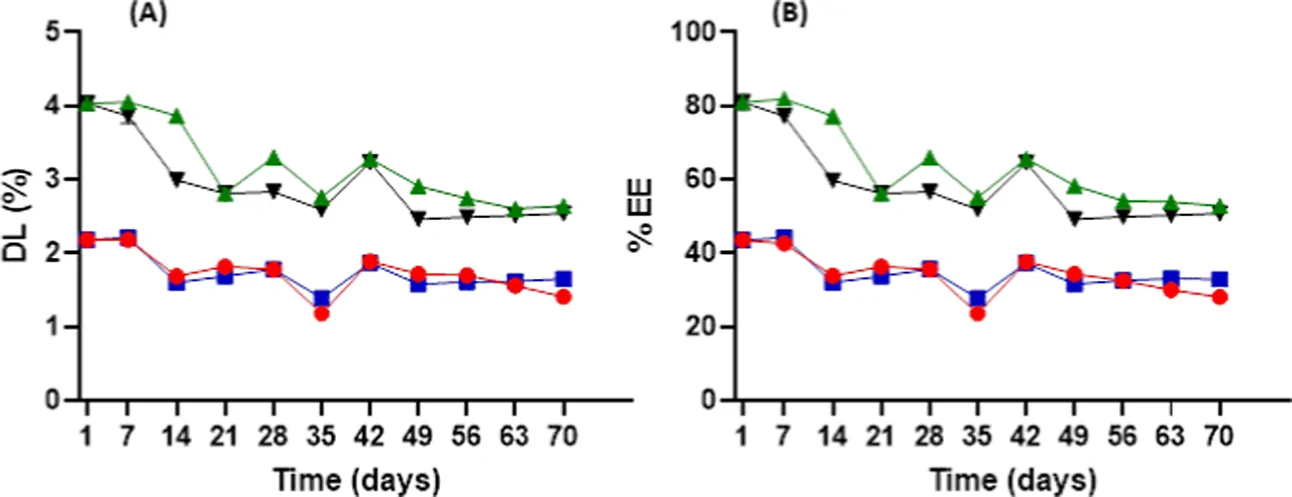
(A) DL of NLC_3_(■(blue square)), NLC_4_ (▲(green triangle)) stored at 4 °C and NLC_3_(●(red circle)), NLC_4_ (▼)­stored at 25 °C. (B) EE of NLC_3_(■(blue square)), NLC_4_ (▲(green triangle)) stored at 4 °C and NLC_3_(●(red circle)), NLC_4_ (▼)­stored at 25 °C.

### 3.2 Morphology

The four NLC formulations are shown in Figure 3. The NLC formulations were globular, homogeneous, and clear. The nonspherical structures are artifacts resulting from sample preparation. The images show that the particle size confirmed the low polydispersity of the NLCs, as indicated by the relatively small PDI values obtained from DLS measurements. Furthermore, the photomicrograph is devoid of aggregates (Figure 3).

**Figure 3:**
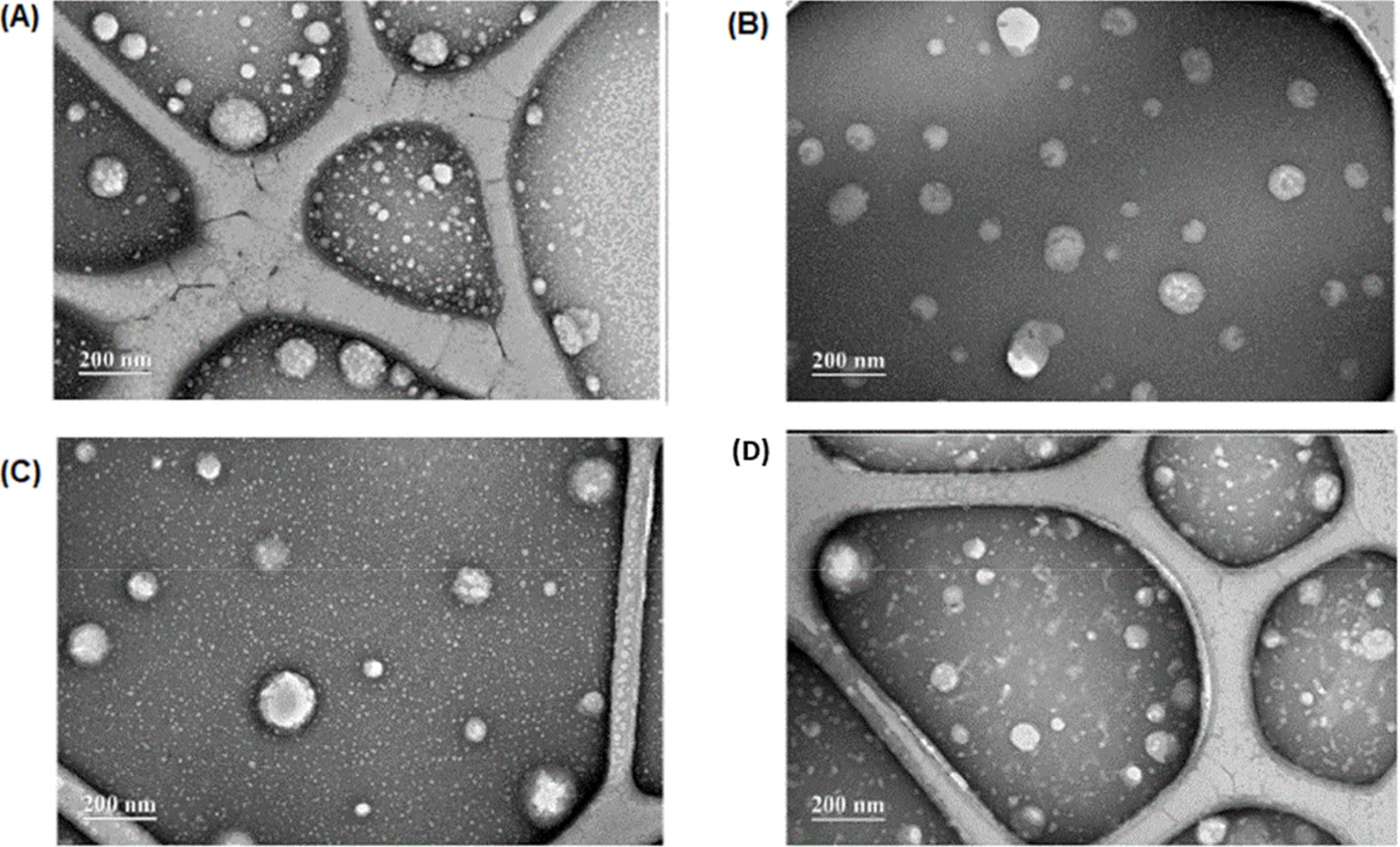
TEM images of NLC (A), NLC_1_ (B), NLC_2_ (C) NLC_3_ and (D) NLC_4_ at magnitude 150000×.

As observed in the images of Figure 4, the particle size confirmed the low polydispersity of NLC_2_, NLC_3_, and NLC_4_, whereas NLC_1_ exhibited high polydispersity. These findings were supported by the relatively low PDI values obtained from DLS measurements, as well as by the relative particle size distribution graphs shown in Figure 4.

**Figure 4:**
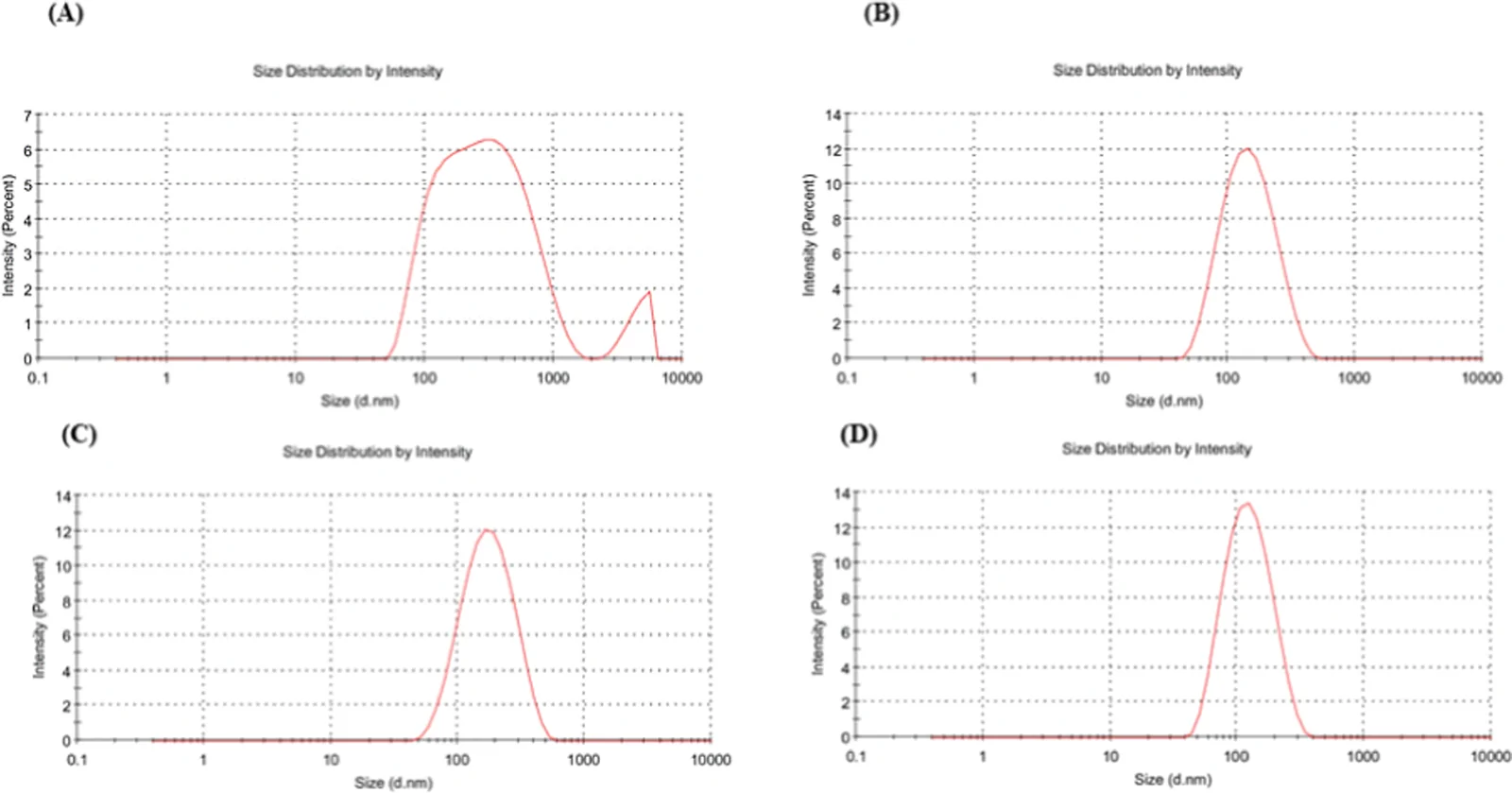
Particle size distribution NLC (A), NLC_1_ (B), NLC_2_ (C) NLC_3_ and (D) NLC_4_ by dynamic light scattering (DLS).

### 3.3 X-ray Diffraction

X-ray diffraction (XRD) analysis was performed to evaluate the physical state of the lipid matrix in the NLCs. The diffractograms of the placebo formulations (NLC_1_ and NLC_2_) and the drug-loaded formulations (NLC_3_ and NLC_4_) are shown in Figure 5. In general, all formulations exhibited four significant peaks: the first at approximately 19.5°, the second at 21.58°, and the third (≈23.3°) and fourth (≈24.9°) peaks, which are almost overlapping, but the angles at which they appear can still be distinguished (Figure 5). The peaks at 19.5° and 23.3° are attributed to residues of tucumã fat, whereas the peaks at 21.58° and 23.90° correspond to residues of carnauba wax.^18^

**Figure 5:**
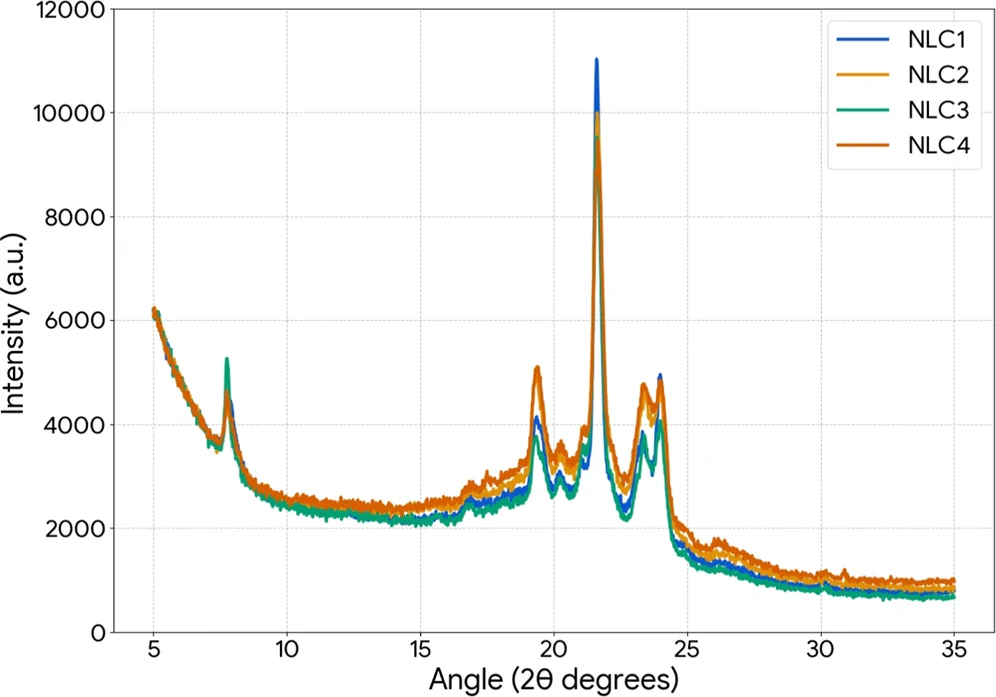
X-ray diffraction spectra of NLC_1_, NLC_2_, NLC_3_ and NLC_4_.

Bragg’s law indicates that the angles 19.5°, 21.58°, 23.3°, and 23.9° correspond to 4.6 Å, 4.1 Å, 3.9 Å, and 3.7 Å, respectively. Based on these values, we determined that the NLCs crystallize in the β (4.6 Å and 3.7 Å) and β′ (4.1 Å and 3.7 Å) polymorphic forms.^23,24^

However, although the X-ray diffraction spectra are very similar, comparative evaluation between the formulations reveals changes in peak intensity. Considering the highest peak, it can be observed that in the placebo formulations the 21.58° peak has higher intensity, with NLC_1_ showing an intensity of 11,022 au and NLC_2_ presenting 9873 au For the ketoconazole-loaded formulations (NLC_3_ and NLC_4_), the differences in peak intensity were less pronounced than in NLC_1_ and NLC_2_, with NLC_3_ showing 9520 au and NLC_4_ 9275 au.

Overall, the diffractograms indicate that all formulations exhibited peaks at the same angles typically observed for carnauba wax and tucumã fat (Figure 5). Moreover, formulations with higher lipid content generally showed higher peak intensities. On the other hand, in the ketoconazole-loaded formulations, peak intensity decreased, and lipid content did not increase peak height, suggesting that ketoconazole interferes with the crystalline structure of the formulations. No peaks characteristic of ketoconazole were observed in the diffractograms, indicating that the drug is molecularly dispersed within the lipid matrix.^25^

### 3.4 Drug Release

In vitro release kinetics were performed to evaluate the release rate of ketoconazole from NLCs compared to free drug (Figure 6). Free ketoconazole reached its maximum release (97%) around 8 h. Meanwhile, NLC_3_ and NLC_4_ showed a release rate of 40% in the first 8 h, reaching 80% of drug release within 60 h of testing, remaining at 80% release until 140 h (Figure 6). The ketoconazole release profile was similar between the two formulations, despite the differences in their composition (Table 1).

**Figure 6:**
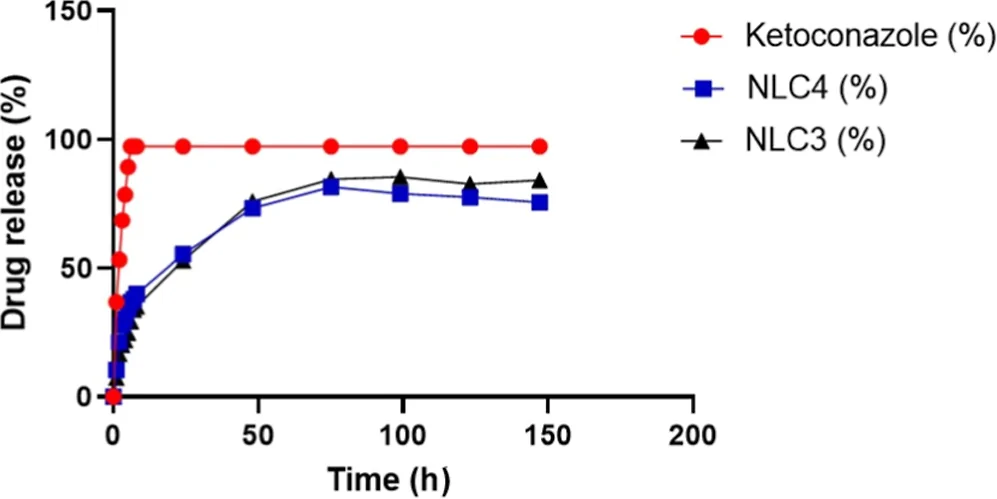
Ketoconazole release profile from NLC_3_ and NLC_4_ by the dialysis bag method.

Table 3 presents the *R* ^2^ values obtained from the drug release analysis data. Using the KinetDS software, *R* ^2^ values were obtained when different mathematical models were tested. The model with *R* ^2^ closest to 1 was the Korsmeyer-Peppas for both NLC_3_ (*R* ^2^ of 0.98) and NLC_4_ (0.97) (Table 3), while other models presented *R* ^2^ less than 0.8.

**Table 3:** Mathematical Models Tested to Investigate the Release Mechanism of Ketoconazole from Nanoparticles

model	*R* ^2^	
	NLC_3_	NLC_4_
zero order	0.7831	0.7000
primary order	0.0806	0.0699
second order	0.0415	0.0415
third order	0.0415	0.0415
Korsmeyer-Peppas	0.9812	0.9746
Hixson-Crowell	0.4857	0.3785
Baker-Lonsdale	0.8767	0.8010

### 3.5 Antifungal Activity

#### 3.5.1 Minimum Inhibitory Concentration

The in vitro antifungal activity of the NLC loaded ketoconazole (NLC_3_ and NLC_4_) and ketoconazole was tested at various concentrations against *Candida albicans* (*C. albicans*) and *Candida tropicalis* (*C. tropicalis*) strains to determine the MIC and MFC (Figures 7, 8 and 9).

**Figure 7:**
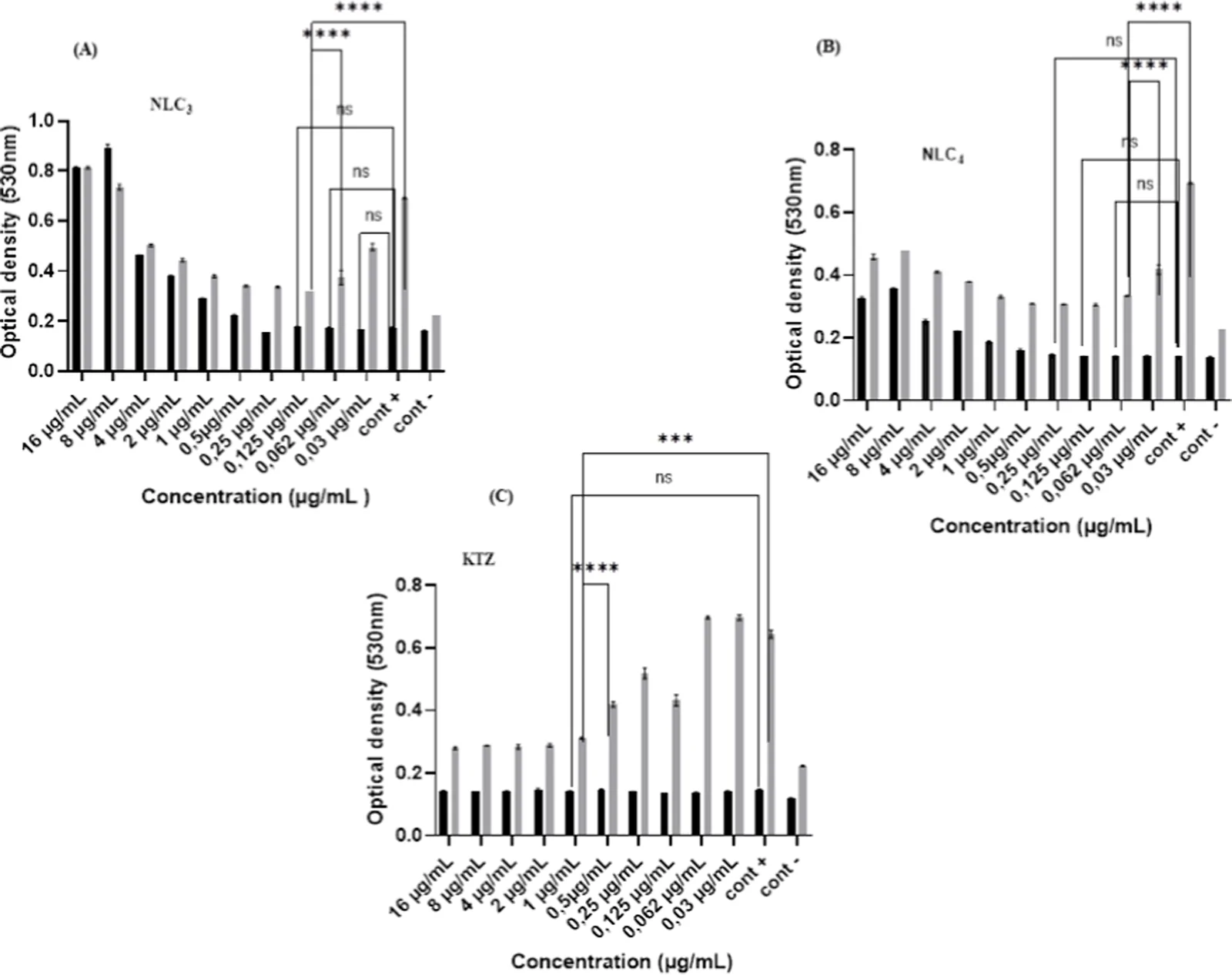
Optical density of *Candida albicans* strains. (A) NLC_3_, (B) NLC_4_ e (C) ketoconazole. Cont+ is growth control. Cont- is sterility control. Results were obtained using ANOVA followed by Tukey’s test. ***p < 0.001, ****p < 0.0001 and n.s. not significant. 24 h (
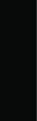
) and 72 h (
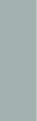
).

**Figure 8:**
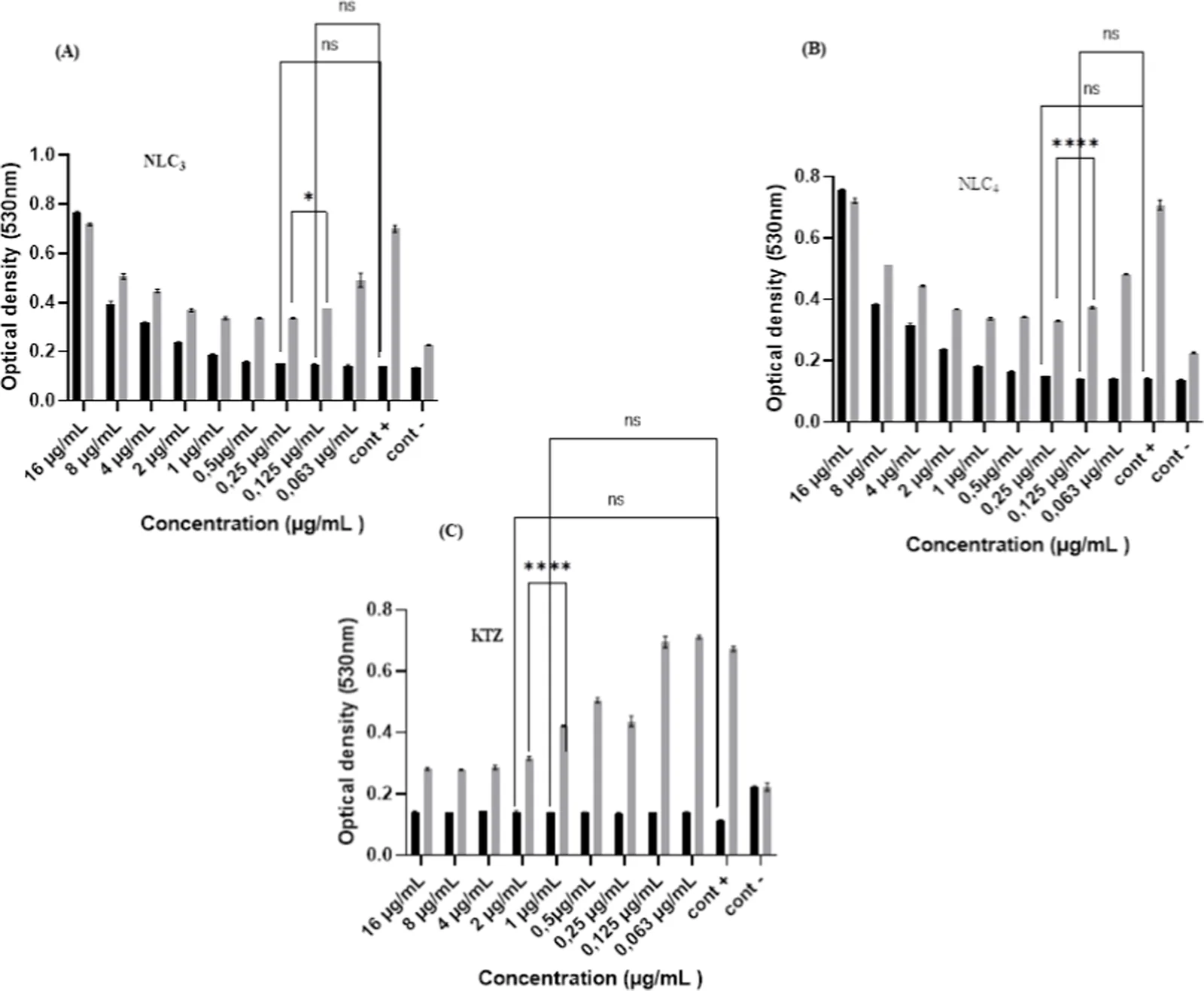
Optical density of *Candida tropicalis* strains. (A) NLC_3_, (B) NLC_4_ e (C) ketoconazole. Cont+ is growth control. Cont- is sterility control. Results were obtained using ANOVA followed by Tukey’s test. ***p < 0.001, ****p < 0.0001 and n.s. not significant. 24 h (
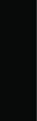
) and 72 h (
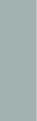
).

**Figure 9:**
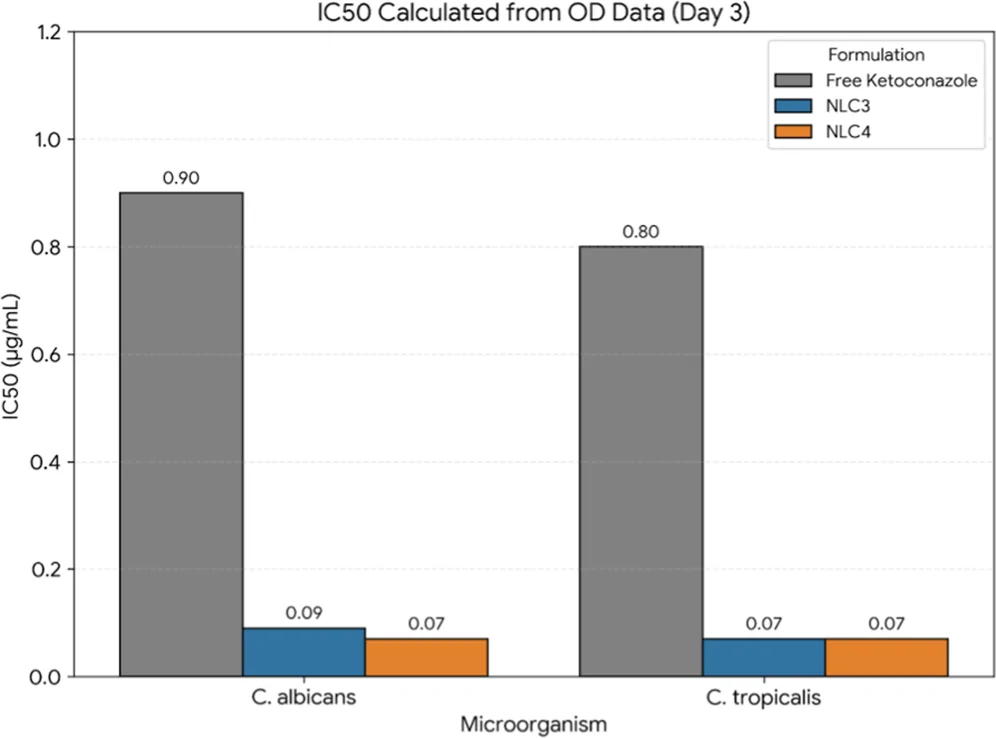
IC50 of ketoconazole solution and carriers NLC_3_ and NLC_4_ to *C. albicans* and *C. tropicalis*.

Figure 7 shows the variation in optical density as a function of the different concentrations of the tested samples. In general, higher optical density corresponds to greater fungal growth; therefore, a decrease in optical density is associated with antifungal activity.^26^ This rule has limitations for samples that can turbify the medium, thereby contributing to an increase in optical density. However, when the optical density increases for this reason, it does not correlate with fungal growth. This occurs with NLC_4_ and NLC_3_, especially at concentrations above 0.5 μg/mL, which show optical density values higher than the positive control (Figures 7 and 8).

From Figure 7, it can be observed that readings taken after 24 h of incubation did not show significant differences from the positive control. However, after 72 h, fungal growth was sufficient to identify the minimum inhibitory concentration (MIC). NLC_3_ and NLC_4_ were able to inhibit the growth of *C. albicans* up to a concentration of 0.062 μg/mL. At a concentration of 0.03 μg/mL, however, neither NLC_3_ nor NLC_4_ could inhibit fungal growth, and a significant increase in optical density was observed (*p* < 0.0001). Accordingly, the MIC of both formulations against *C. albicans* was 0.062 μg/mL. For ketoconazole, Figure 7C shows a MIC of 1 μg/mL.

When tested against *C. tropicalis*, fungal growth after 24 h was also not significant, but the MIC of the samples could be determined after 72 h. Figure 8 shows that the MIC values for NLC_3_, NLC_4_, and ketoconazole were 0.25 μg/mL, 0.25 μg/mL, and 2 μg/mL, respectively.

Finally, the IC_50_ of the formulations was approximately 10 times lower than that of free ketoconazole, as shown in Figure 9.

#### 3.5.2 Determination of Minimum Fungicidal Concentration

The minimum fungicidal concentration is the lowest concentration of the substance in which no visible growth of subculture occurs.^26^ The MFC of NLC3, NLC4, and ketoconazole was 0.25 μg/mL, 0.25 μg/mL, and 2 μg/mL, respectively, for both *C. albicans* and *C. tropicalis* (Figure 10).

**Figure 10:**
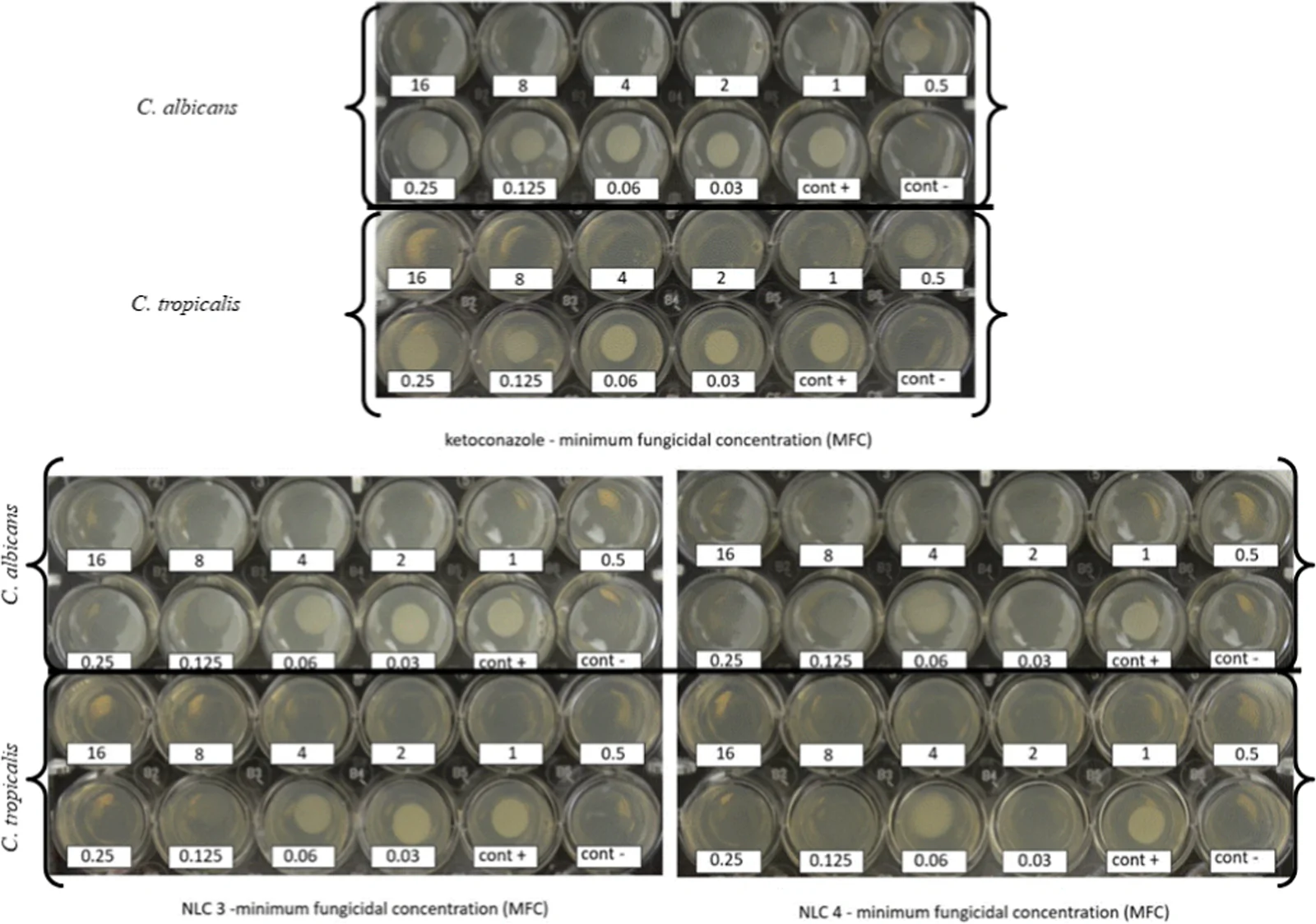
Minimum fungicidal concentration of ketoconazole solution and carriers NLC_3_ and NLC_4_ to *C. albicans* and *C. tropicalis*.

The minimum fungicidal concentration showed that the incorporation of the drug into the carriers improved its antifungal activity when compared with ketoconazole in solution, where free ketoconazole had a MFC of 2 μg/mL and the carriers NLC_3_ and NLC_4_ had a MFC of 0.25 μg/mL (Figure 10).

## 4 Discussion

NLCs obtained from tucuma fat were prepared to observe their ability to encapsulate ketoconazole and remain stable, as well as to verify the effectiveness of these formulations against *Candida* species. The use of lipid nanoparticles as antifungal drug carriers is based on the advantages that their small size offers for drug delivery, such as greater ease of penetration through the skin, when compared to conventional dosage forms such as gels, emulsions, pastes, solutions, ointments, among others.^27^

In this context, NLC size, as well as polydispersity index (PDI) are critical nanoparticle quality parameters, important for assessing their physical stability.^28^ PDI values reflect the degree of dispersion among nanoparticle sizes. Specifically, PDI values equal to or below 0.1 indicate highly monodisperse systems, while PDI values between 0.1 and 0.4 indicate slightly polydisperse.^20^In general terms, the lower the PDI value, the lower the degree of polydispersity.^29^ NLC1 was the most polydisperse formulation (PDI between 0.4 and 0.6). In absolute terms, NLC1 contains the highest amount of lipids. The lipids contribute to increased viscosity of the oil phase and greater hydrophobicity, which hinders emulsification and results in formulations with larger particle sizes and higher PDI values.^29,30^

Regarding the PDI, no significant correlation was observed between storage time and changes in PDI values for any of the formulations. The same was observed for particle size and zeta potential. These results indicate that the nanoparticles did not undergo physical instability processes commonly observed in this type of system. The stability of solid lipid nanoparticles can be understood through the DLVO theory (from the Derjaguin–Landau–Verwey–Overbeek initials). The DLVO theory is widely used to describe the stability of colloidal systems, which are, by definition, composed of microscopic particles dispersed in a medium. The duration for which these particles remain dispersed serves as a measure of stability. According to the DLVO theory, the interaction between two approaching nanoparticles can be described as the balance between an attractive interaction potential, arising from hydrophobic van der Waals forces, and a repulsive electrostatic interaction potential, originating from ionizable groups on the nanoparticle surface. NLCs are in a constant state of Brownian motion, and collisions between nanoparticles are common. This collision can lead to aggregation (when attractive forces dominate) and “adhesion” of the nanoparticles.^30–33^ Variations in particle size and PDI values may indicate processes of physicochemical instability in the formulation. Extrinsic factors, such as storage temperature, affect the susceptibility of formulations to maintaining physical stability. Exposure of NLCs to light and/or increased temperature contributes to the destabilization of formulations.^34^ In particular, increased temperature can lead to increased particle size and, consequently, variations in PDI.^31–33^ Overall, the PDI and particle size data indicate that the samples stored at room temperature and at 8 °C maintained their physical stability, showing no signs of particle aggregation or coalescence. On the other hand, the same cannot be said for the formulations stored at 45 °C.

Regarding the physical stability of the formulations, zeta potential is an important parameter in the characterization of emulsified systems. Zeta potential values above ±30 mW are preferred for emulsions because they are assumed to be an adequate value to ensure repulsion between the particles of the dispersed phase and hinder coalescence.^34,35^ However, in our study, we observed zeta potentials below ±30 mV, and the system was unstable. Thus, we hypothesize that the stability is attributed to the action of Pluronic F127, which acts as a steric stabilizer through the adsorption and incorporation of its hydrophobic PPO (polypropylene oxide) blocks onto the surface of the nanoparticles, justifying the zeta potential values below ±30 mV.^36–38^

The shelf life of pharmaceutical formulations is a critical quality attribute that must be established through systematic stability studies, as it defines the period during which a product remains within its predetermined specifications of identity, purity, potency, and performance under recommended storage conditions.^39^ Our stability data show information about physicochemical stability, such as the significant decrease in EE and DL values after 2 weeks of storage. This occurred with both formulations stored at temperatures of 4 and 25 °C. NLC_3_ and NLC_4_ both underwent a decrease in EE and DL during storage, possibly due to crystal reorganization. It is possible that the drug was expelled during storage. Such release may result from polymorphic transformations of triglycerides in the lipid phase. Polymorphic transformation is an irreversible process in which a lipid system transitions from a less stable form to a more stable one, influenced by both temperature and time. Under constant temperature conditions, the α and β′ forms of triglycerides can gradually convert into the β form.^24,40^ However, monitoring the crystalline structure of the NLCs is required to confirm whether crystalline transformations occur.

This study also evaluated the release of ketoconazole from NLCs. This release study is a complex mechanism involving a combination of diffusion and erosion. This explains why the Korsmeyer-Peppas model presented the highest *R* ^2^. The flexibility predicted by this model can explain the complex mechanism of drug release from NLCs. In any case, NLCs exhibit a two-stage drug release pattern: an initial rapid release followed by sustained release at a constant rate. The initial release is influenced by the presence of liquid lipids in the lipid matrix.^41,42^

Finally, the antifungal efficacy of NLCs was tested against *Candida* strains. Candida are yeasts of clinical and epidemiological importance that can cause medical issues in the mucous membranes, gastrointestinal tract, and urinary system, and are more prevalent in immunocompromised individuals, pregnant women, and the elderly. Among the clinically important Candida species are *C. albicans* and *C. tropicalis*.^43^ Ketoconazole acts against these yeasts by inhibiting ergosterol synthesis, which causes alterations in the cell membrane, cell lysis, and necrosis. Nanoencapsulation of ketoconazole in biocompatible lipid nanoparticles with prolonged release results in improved solubility and a larger surface area, resulting in improved efficacy.^44^

## 5 Conclusions

Nanostructured lipid carriers containing tucumã fat were produced in order to evaluate this lipid matrix as a carrier for antifungal drugs, using ketoconazole as a model drug. The results obtained allowed us to conclude that tucumã NLCs potentiated the antifungal action of ketoconazole, and the NLCs presented better values for minimum inhibitory concentration and minimum antifungal concentration. However, stability studies indicated possible polymorphic transformations during storage, which resulted in the expulsion of the drug. This point needs to be further investigated by monitoring the crystalline structure of the NLCs during storage.

Finally, although it presented limitations, we were able to build the bioactive platform using tucumã butter, and future steps would be to consider incorporating this system into a pharmaceutical platform that facilitates its topical application. Gels, hydrogels, or films are options for pharmaceutical forms that could carry tucumã NLCs, and from there, design the use of these formulations in the treatment of cutaneous, vaginal, and/or oral mucosal mycoses.

## Supplementary Material



## Notes

The Supporting Information file presents the quality control data provided by the manufacturers of the raw materials used in this study (PDF)

## Abbreviations

NLCs: nanostructured lipid carriers
EE: encapsulation efficiency
DL: drug loading
SLNs: solid-lipid nanoparticles
BCS: biopharmaceutical classification system
HPH: high-pressure homogenization
PDI: polydispersity index
HPLC: high-performance liquid chromatography
TEM: transmission electron microscopy
CI: crystallinity index
DSC: differential scanning calorimetry
MIC: minimum inhibitory concentration
MFC: determination of minimum fungicidal concentration
OD: optical densities
DLVO: Derjaguin–Landau–Verwey–Overbeek.
