# Supplementary material for: Tucuma Fat-Based Nanostructured Lipid Carriers: Physical Stability and Antifungal Activity
Source: ACS Omega. 2026 May 6;11(19):27958–69. doi: 10.1021/acsomega.5c11826 (PMC13557264; doi:10.1021/acsomega.5c11826)
Supplement: Supplementary file 1 [file ao5c11826_si_001.pdf]

Tucuma Fat-Based Nanostructured Lipid Carriers: Physical stability and antifungal activity

Antônio Taylon Aguiar Gomes[a], Rayanne Rocha Pereira[b], Lindalva Maria de Meneses Costa Ferreira[a], Naila Ferreira da Cruz[c], Desireé Gyles Lynch,[d] Patrícia Fagundes da Costa[c], Claudio Guedes Salgado[c], José Otávio Carréra Silva-Júnior[a] and Roseane Maria Ribeiro-Costa [a].*

[a]Institute of Health Sciences, Federal University of Pará, Belém-PA, 66075-110, Brazil.

[b]Institute of Collective Health, Federal University of Western Pará, Santarém-PA, 68035-110, Brazil.

[c]Institute of Biological Sciences, Federal University of Pará, Belém-PA, 66075-110, Brazil.

[d]University of Technology, Jamaica, College of Health Sciences, School of Pharmacy, Jamaica, Kingston 6.

[*]Corresponding author: E-mail: rmrc@ufpa.br, Tel/Fax numbers: ++ 5591 3201-7203/ 3201-7201.

Supplementar Material 1- Quality control reports for the raw materials used

Table S1: Results described in the carnauba wax analysis report, according to the supplier, GM Ceras.

Análise	Especificações	Resultado
Cor	Amarela	Ok
Odor	Característico	Ok
Aparência	Escamas	Ok
Ponto de fusão (°C)	81 – 86	83,00
Índice de Saponificação (mg KOH / g)	78 – 88	86,40
% Cinzas	0,00 – 0,12	0,10
Úmidade	0,00 – 0,50	0,49
Índice de Ácidez (mg KOH / g)	2,0 – 6,0	4,50

Table S2: Results described in the ketoconazole analysis report provided by Natural Pharma.

Análise	Especificações	Resultado
Identificação	Conforme USP	De acordo
Rotação especifica	De -1,0 a +1,0°	0°
Ponto de fusão	148 - 152°C	148 - 150°
Perda por secagem	Máximo de 0,50%	0,1%
Resíduo de ignação	Máximo de 0,10%	0,05%
Doseamento	98,0 – 102,0%	99,52%
Metais Pesados	Máximo de 0,002%	< 0,002%
Pureza cromatográfica	Máximo de 2,0%	De acordo
Acetona	Máximo de 0,5%	0,0%
Acetoacetato	Máximo de 0,5%	0,22%
Dimetilsulfóxido	Máximo de 0,5%	0,0%

Ministério da Saúde

FIOCRUZ

Fundação Oswaldo Cruz

Instituto Nacional de Controle de Qualidade em Saúde

INCQS

CERTIFICADO

SUBSTÂNCIA QUÍMICA DE REFERÊNCIA DA FARMACOPÉIA BRASILEIRA

SQRFB

cetoconazol

Lote: 1033

Descrição: pó branco a quase branco.

Teor: 99,8%, calculado em relação à substância dessecada, determinado por titulação potenciométrica com ácido perclórico 0,1M.

Armazenamento: conservar o frasco bem fechado, protegido da luz e em refrigerador (em temperatura entre 2ºC e 8ºC).

Agência Nacional de Vigilância Sanitária

FARMACOPÉIA BRASILEIRA

Av. Brasil, 4365 Manguinhos CEP 21040-900 Rio de Janeiro RJ Brasil Tel (21) 3865-5151 Fax (21) 2290-0915

www.incqs.fiocruz.br

Figure S1: Analysis report for ketoconazole provided by the Oswaldo Cruz Foundation.

Table S3: **Results described in the tucumã fat analysis report provided by Amazon oil.**

DADOS FÍSICO-QUIMICOS DA MANTEIGA DE TUCUMÃ		
**CARACTERÍSTICA**	**UNIDADE**	**ESPECIFICAÇÃO**
Aparência (25Cº)	----	Sólido
Cor	----	Branco para bege
Odor	----	Característico
Índice de acidez	mg KOH/g	< 20,0
Índice de peróxido	meq O_2/kg	< 15,0
Índice de iodo	gI_2/100g	10 – 30
Índice de saponificação	mgKOH/g	230 – 250
Densidade	25ºC g/ml	0,967
Matéria insaponificável (bioativos)	%	1,4
Ponto de fusão	°C	30 – 32
**COMPOSIÇÃO DOS ÁCIDOS GRAXOS**		
Ácido caprílico (C 6:0)	%	0,5 - 1,5
Ácido cáprico (C 8:0)	%	1,0 - 2,0
Ácido láurico (C 12:0)	%	44 - 55
Ácido mirístico (C 14:0)	%	22 ,0- 30,0
Ácido palmítico (16:0)	%	5,0 - 10,0
Ácido esteárico (18:0)	%	1,0 - 3,0
Ácido oleíco (18:1 Omega 9)	%	8,0 - 10,0
Ácido linolênico (C 18:3 Omega 3)	%	2,0 - 5,0
Saturado	%	97
Insaturado	%	3
